# Full-Scale Use of Microwave Heating in Construction of Longitudinal Joints and Crack Healing in Asphalt Pavements

**DOI:** 10.3390/ma14185159

**Published:** 2021-09-08

**Authors:** Maciej Maliszewski, Adam Zofka, Dominika Maliszewska, Dariusz Sybilski, Bartłomiej Salski, Tomasz Karpisz, Rafał Rembelski

**Affiliations:** 1 Pavement Technology Division, Road and Bridge Research Institute, 03-302 Warsaw, Poland; azofka@ibdim.edu.pl (A.Z.); dmaliszewska@ibdim.edu.pl (D.M.); d.sybilski@ibdim.edu.pl (D.S.); rrembelski@ibdim.edu.pl (R.R.); 2 The Institute of Radioelectronics and Multimedia Technology, The Faculty of Electronics and Information Technology, Warsaw Technical University, 00-665 Warsaw, Poland; bsalski@ire.pw.edu.pl (B.S.); tkarpisz@ire.pw.edu.pl (T.K.)

**Keywords:** asphalt mix, longitudinal joints, microwave heating, dielectric heating, mechanical properties, physical properties, asphalt pavement, self-healing, asphalt pavement repair, cracks-healing

## Abstract

Asphalt pavement construction technology is an industry branch that undergoes constant development. Analyzing the directions of the development, one can divide it into two mainstreams: the development of roadworks equipment and the development of roadworks technology. Microwave heating technique has been mentioned in the road industry from the early ‘70s, but research records from practical full-scale use are very rare. This article presents the evaluation of the possible use of microwave heating technique during a particular aspect of the construction process, namely, the formation of longitudinal joints and the potential repair process of the cracked asphalt pavement. Research results showed that joints constructed using microwave-assisted heating performed the same or even better with regards to tensile characteristics comparing to other techniques. Also, the highest level of compaction was reached among the other tested techniques applied to the wearing course level. The second part of the research experiment showed the large potential of the microwave crack healing technique. The asphalt pavement was healed on its full depth of 10 cm with the single healing operation applied. Although some limitations may occur in the practical use of microwave heating, the test results suggest that it is a very promising technique and should be further developed (for, e.g., shielding concerns, electricity supply). The microwave heating technique is powered with electricity, which is important when there is a constant need for further reductions of CO_2_ emissions. It can be reached in parallel with clean energy or clean electricity sources.

## 1. Introduction

Proper paving of the asphalt layer is a very complex process requiring controlling and synchronizing of many separate processes to gain successful and durable effect. Three main processes in the paving of asphalt layer can be addressed, i.e., preparation of durable and bearable subgrade for paving asphalt layer, hot mix asphalt production and transport, and forming of asphalt layers and compaction. One of the inevitable stages of the laying and compaction process is the formation of longitudinal and transversal joints. Laying of the monolith, a seamless asphalt layer with no vertical discontinuities, would be the most desirable method, but it is not possible in 75% of cases [1]. Unfortunately, it requires formation of the joints or wedges that are of worse quality in terms of pavement properties than monolith multilayered structure [2]. Because the pavement joint area is usually characterized by a lowered compaction degree, asphalt mix segregation or discontinuity, it is vulnerable to premature deterioration. Thus, the joint area in typical, dense graded asphalt pavement is susceptible to premature cracking [3] or aggregates evacuation caused by factors including water impact, freeze and thaw cycles or bitumen aging [4,5]. After the formation of discontinuity, a repair process is needed. In the case of asphalt pavements, the repair is usually done using hot-applied joint sealants or cold-applied joint sealants and materials [6].

There are several classical methods of constructing longitudinal joints. A brief review of pavement longitudinal joint-forming technology together with the pros and cons was presented in Table 1 [7,8].

Proper strategy and technology of asphalt layer paving should ensure good quality of longitudinal joints and minimize the potential of discontinuity. As the seamless joint-forming technique is unlikely to be used in most cases, “hot by cold” technology must be used. Cold layer, namely, its edge to which next layer will be paved, must be properly prepared before laying next hot mix layer. There are several ways to prepare the so-called “cold edge”. There are forming techniques, sealing techniques and heating techniques. The first group briefly consists of the following:

- skewed profiling (60°), formed by the roller compactor (or paver) equipped with the edge forming plate,
- vertical profiling, formed with the use of knife installed on roller compactor; formed after compaction of asphalt layer when it cools down below compaction temperature,
- different wedge compactors for forming the longitudinal joints,
- a different strategy of asphalt paving and compaction order, in particular, the following:
  ◦ use of adequate thickness of asphalt layer, which cannot be thinner than cold lane after compaction, because it may lead to under compaction issue,
  ◦ use of the paver controlled with an averaging ski, helping to synchronize layers thickness,
  ◦ establishing of steel or rubber roller compactor passes over the joint,
  ◦ the finish of compaction work before reaching layer minimum compaction temperature.

Second group, the use of additional sealing material, is also very popular in Polish road construction practice. The following materials can be used:

- additional tack-coat application over the cold lane edge (asphalt emulsion, hot asphalt or bituminous paste),
- additional sealing material, e.g., bituminous tape, bituminous paste or bituminous sealant.

The last group is still not popular in Polish road engineering. The construction of longitudinal joints with the following techniques is possible:

- reheating of cold lane edge before or after application of hot layer using infrared heaters [9,10],
- reheating of cold lane edge using induction heating [11],
- reheating of cold lane edge using microwave applicator.

Akpir [12] names particular problems occurring during the formation of joints: low compaction, irregularity and segregation of aggregate. These problems induce locally increased water permeability of the structure, thus decreasing water and frost resistance. While some of the technologies are bringing promising results, e.g., infrared heating technology can help increasing compaction ratio, some authors claim that microwave heating potentially could improve properties of the joint [13]. IBDiM and the industry partner QWED sp. z o.o. finished a project named NGAM2, cofounded by the Polish National Center for Research and Development [NCBR, grant no. PBS2/B3/19/2013]. One of the main aims of this project was to evaluate the possibility and assess the effectiveness of the use of microwave technology to improve the quality of road works. Basing on the project results, the PhD thesis and monography was published [14]. This article focuses on the research concerning hot by a cold joint-forming technique using a standard untreated technique (vertical and skewed joint), additional material sealing as well as additional joint treatment.

**Figure 1:**
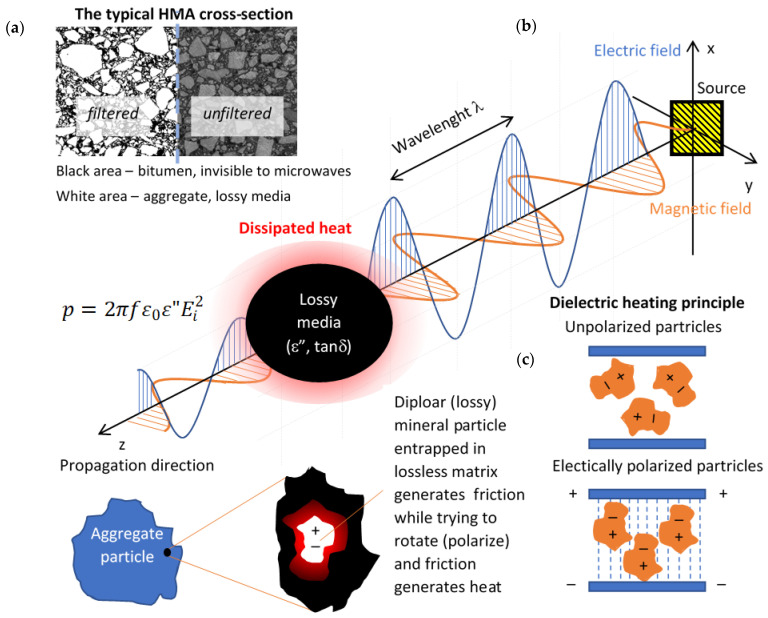
(**a**) HMA cross-section—white regions are capable of being dielectrically heated; (**b**) Microwave heating scheme (p—power density in Watts/m^3^, ε_0_—electric permittivity of the free-space, ε″—dielectric loss constant, E_i_^2^—mean square electric field intensity); (**c**) dielectric heating principle.

Microwave heating (also known as dielectric heating) fundamentals are described using theory formulated by Maxwell [15], which has been described using differential equations [16,17] and is well defined in the literature, e.g. [18]. The dielectric heating uses energy transfer in contrast to traditional heating, which only uses the heat transfer by radiation, convection or conduction. Dielectric heating is of volumetric type and thus achieves higher heating rates than conventional convection or conduction heating, giving overall reduction of overall processing time [19,20]. But there are some restraints in using dielectric heating, which come from dielectric properties of materials. Not all media are capable of being heated dielectrically, as some of them are transparent to dielectric heating. The dielectric constant ε′, loss tangent tan δ and dielectric loss ε″ are the parameters that describe capabilities of media to be dielectrically heated. The ideological scheme of dielectric heating of hot-mix asphalt is presented in Figure 1.

Regarding asphalt pavements, one of the asphalt constituent materials, the bitumen is almost invisible to dielectric heating while the main constituent, the aggregate, is dielectrically heatable [21]. Aggregates typically used in asphalt production are of a different mineral composition resulting from the locally available rock material. There is a growing number of research articles devoted to microwave heating of pure asphalt mixes. Researchers have tested the microwave heating influence on asphalt properties [22,23,24], the possibility of assisting the recycling process [21,25,26], the possibility of asphalt mix self-healing [27,28,29] or healing [30,31]. Other researchers are modeling the heating influence and healing potential of microwaves or induction heating on asphalt mix properties [32,33]. Recent research is directed on the use of special aggregates or steel slags [34,35,36,37], modified bitumen [38] or additives and admixes [39,40,41], which help to improve the asphalt dielectric heating ability or healing potential [42]. 

In this study, there were no extra constituent materials added nor applied, and asphalt mixes were formulated using the typical aggregate available on the Polish market. Presented test results are not focused on the properties of the asphalt itself but the performance-related properties of asphalt longitudinal joints. In addition the full-scale, crack repair was evaluated as well.

## 2. Materials and Methods

### 2.1. Testing Program

The presented research is a part of the research program that consisted of three phases:

- Phase 1––the laboratory evaluation of dielectric properties of asphalt materials and constituting materials (presented in [43]),
- Phase 2––the laboratory evaluation of dielectric heating on the properties of asphalt, aggregate and bitumen,
- Phase 3––the real-scale experiment of the use of dielectric heating during construction and repair process of the asphalt pavement.

There were several prototype devices designed within the scope of the research program, different resonators for evaluation of dielectric properties of asphalt and its constituents, microwave applicator for applying the dielectric heating to the test specimens and the prototype microwave applicator for the treatment of asphalt pavement during construction and repair process. This article focuses on the third phase of the research program, the real-scale treatment of longitudinal joints and cracks. For the durability considerations, there were several testing techniques used considering mechanical strength of asphalt mixes sampled from the joint or crack area. Strength parameters were assessed directly on testing specimens in different temperatures that are likely to occur in natural conditions. To assess the influence of microwave heating on the asphalt pavement durability, there were also some material (bitumen) aging tests performed. These tests indicate predicted pavement cracking susceptibility performance.

### 2.2. Design of Experiment, Scope of Research, Test Section

There were three main objectives of the testing program presented in this paper:

- to evaluate if microwave-assisted joint construction technique has advantages over traditional methods of joint construction,
- to evaluate the effectiveness of crack repair using microwave heating technique,
- to consider if dielectric heating causes more severe damage to the bituminous binder than traditional heating.

The whole experiment was meant to evaluate the in-situ effectiveness of the microwave applicator designed within the scope of NGAM2 project [44]. This article focuses on the performance evaluation of chosen joint construction techniques commonly used in Poland, together with an innovative technique of microwave joint treatment. The full spectrum of joint techniques used in road practice was chosen. There were three groups of joint construction technologies and one crack repair technique evaluated, and their description and abbreviation used in this article are shown in Table 2. It is worth mentioning that direct comparison is possible between VJ, IR (formally VJ + IR) and MW (formally VJ + MW) because all these types of joints are formed in the vertical plane. Sealed type joints, namely T1, T2, P1 and P2 (formally VJ + T1, VJ + T2, VJ + P1 and VJ + P2) were of similar geometry but additional material was added, which affects the joint properties. The performance of joint construction techniques and crack repair was limited to direct mechanical tests on specimens cored from joints, density measurements, as well as tests of bitumen prepared in laboratory conditions.

To summarize, the independent variables were the joint-forming techniques and bitumen aging techniques, while the dependent variables were the strength and density characteristics of joints and bitumen parameters calculated on basis of dynamic shear rheometer analysis.

**Figure 2:**
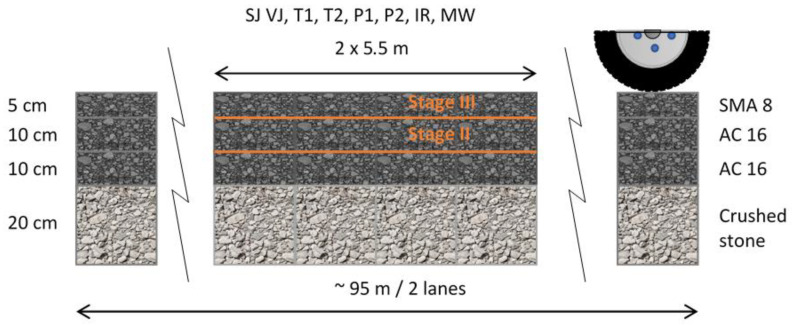
The schematic longitudinal cross-section of the longitudinal joints construction test section.

The whole experiment was performed in operational conditions, on a real-scale road test section, constructed under controlled conditions, using an ordinary roadworks equipment. The test section was a 2-lane, 95 m long full-scale asphalt pavement, comprising crushed stone sub-base (20 cm thick), two AC 0/16 mm layers (each 10 cm thick) and SMA 0/8 mm layer (5 cm thick). AC 16 asphalt mix was used to pave the base course and binder course, while SMA 8 mix was used to pave wearing course. The test section was divided into subsections, which were doubled for each joint construction technique. Figure 2 presents the schema of the test section.

### 2.3. Materials

Test section was made of asphalt layers. Basic physical, functional and dielectric properties of constituent materials used in asphalt mix design are presented in Table A1, Table A2 and Table A3 (Appendix A). Properties and respective standards were also presented.

Mix designs used to form asphalt layers are presented in Table 3. Pavement lanes were intentionally paved in two separate processes. Hot lane was paved next to the lane that has already cooled down. There was different technology for constructing longitudinal joints used. Longitudinal joints were formed between binder courses (designation-II) and between wearing courses (designation-III). Both, the constituent materials as well as the layer configurations were described in Table 4.

There were four types of sealing materials used in this experiment. These were two sealing tapes (T1 and T2) and two sealing pastes (designated as P1 and P2). Properties of these materials are presented in Table 5.

### 2.4. Test Specimens and Test Methods

**Figure 3:**
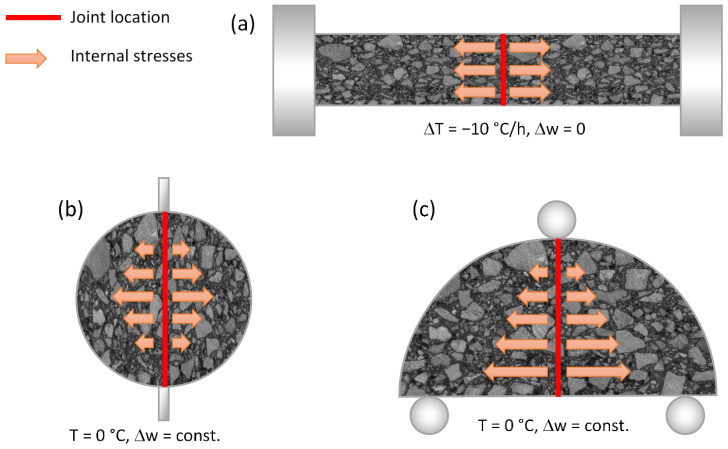
(**a**) Joint location in TSRST specimen (deflection rate −Δw = 0 mm/min); (**b**) Joint location in ITS specimen (deflection rate −Δw = 50 ± 2 mm/min); (**c**) Joint location in SCB specimen (deflection rate −Δw = 5 ± 0.2 mm/min).

Asphalt mixture specimens were cored form the real-scale asphalt pavement using a diamond coring machine (100 mm and 150 mm diameter cylindrical specimens) or cut from pavement using a saw (100 mm × 100 mm × 200 mm perpendicular specimens). On every type of longitudinal joint section there were at least five specimens taken. Specimens were extracted from the pavement in two stages, stage II and stage III separately (see Table 4). After the extraction process, the longitudinal joint (or healed crack) was located in the middle of the specimen, shown in Figure 3. In the laboratory, specimens were cleaned and cut on the precision saw to level the specimens’ surfaces. Perpendicular specimens and 150 mm cylindrical specimens were additionally cut in half to gain proper specimen size described in Table 6. The conditioning process was adequate to the testing protocol described in Table 6. 

Bitumen samples were supplied by bitumen refinery and prepared in the laboratory and subjected to both standard aging protocol [45] (Rotating Thin Film Oven Test–RTFOT + Pressure Aged Vessel–PAV) and the microwave conditioning process described later.

It is worth mentioning that some problems were occurring during the operations on specimens. Not all specimens that were meant to be further evaluated and tested went positively through the whole process of cutting, reducing and conditioning. Specimens sealed with P1 solvent-based paste after sampling the joint appeared to be very soft (gently movable) and the soft scent of solvent was present. It means that not the whole solvent evaporated after the installation process. This “softness” of the specimen resulted in an overall lowering of the stiffness modulus and strength in different test temperatures.

To evaluate the performance of joints, there were two main measuring techniques used. The first technique used was the mechanical tension performance measurements, which were meant to confirm the influence of different joint construction techniques on adhesion and cohesion, generally the bonding quality of the joint. The second technique was the compaction degree measurement, which was meant to evaluate the most important factor, the quality of roadworks. Mechanical performance was evaluated in terms of tensile strength in different testing modes and temperatures. There were three testing techniques used: indirect tension (IT-CY [46]), semi-circular bending (SCB [47]) and thermal stress restrained tension test (TSRST [48]), so mechanical tests were based on standard test protocols. Compaction degree was calculated based on the bulk density test method saturated surface dry (ssd) and reference Marshall bulk density (ssd). Detailed conditions of the tests and specimen sizes are presented in Table 6.

Direct tension in the thermal cracking test was calculated using Equation (1) (Figure 3a).

(1) $$\sigma_{max}=\frac{F_{max}}{b\cdot t}$$

where *σ_max_*—tensile strength in megapascals, *F_max_*—ultimate force in Newtons, *b*—specimen width in millimeters, *t*—thickness of the specimen in millimeters.

Indirect tensile strength was calculated using the Equation (2) (Figure 3b).

(2) $$\;ITS=\frac{2\cdot P}{\pi \cdot D\cdot H}$$

where *ITS*—indirect tensile strength expressed in megapascals, *P*—ultimate force, expressed in Newtons, *D*—diameter of the cylindrical specimen expressed in millimeters, *H*—the height of the specimen expressed in millimeters.

Bending tensile strength SCB was calculated using the Equation (3) (Figure 3c).

(3) $$\sigma_{max}=\frac{4.263\cdot F_{max}}{D\cdot t}$$

where *σ_max_*—tensile strength in megapascals, *F_max_*—ultimate force in Newtons, *D*—specimen diameter in millimeters, *t*—thickness of the specimen in millimeters.

Compaction degree was calculated according to Equation (4).

(4) $$P=\frac{B_{specimen}}{B_{reference}}$$

The schema of the joint locations within the test specimens is shown in Figure 3.

The durability of asphalt pavements can be expressed using different characteristics, but there are two dominating factors: rutting and cracking. In addition to the above mentioned mechanical tests describing the susceptibility to cracking, there was also a wider analysis performed on bitumen properties. There are two parameters [49] based on bitumen properties, which were found to correlate with field observed cracking of asphalt pavements [50]. The first value is the G-R parameter, which describes the position of the bitumen master curve, which is tested at 15 °C and the shearing speed of 0.005 rad/s. The parameter’s values classified below 180 kPa are found to be of low risk of asphalt pavement cracking, while values over 450 kPa are pointing the high risk of the pavement cracking. The second parameter is the R-value, which describes the master curve shape. It is calculated based on the bitumen DSR test results, where the intersection point of glassy modulus line Gg (assumed 2.5 GPa) and asymptotic line to the initial phase of complex modulus vs. phase angle plot is determined. Rheological index R-value is the difference between glassy modulus Gg and complex modulus in the transition frequency. Bitumen R-value exceeding 3 is estimating the high potential risk of the pavement cracking.

In this analysis, there were 2 bitumen types tested, the 35/50 and PMB 45/80–55. The processing cycle consisted of RTFOT (referred to as 1R), PAV (referred to as 1P) and microwave conditioning process (M:800–900). RTFOT is a well-known standard processes for short-term aging of bitumen (simulates manufacturing and hot-mix placement aging). The testing specimens of 35 g are poured in to the glass cylinders and placed in special rolling rack in the temperature controlled chamber at 163 °C for 85 min, with airflow into glass bottles of 4000 mL/min). PAV aging is known as long-term bitumen aging (simulates 7 to 10 years of in-service conditions). The bitumen samples are kept on stainless steel pans in temperature of 100 °C, pressure of 2.1 MPa for 20 h. 

As regards the microwave heating, authors of the publication [45] proved that 11 g bitumen specimen subjected for 7 h 20 min of microwave heating combining the temperature of 143 °C, different fan speed, 3.08 MPa air pressure and 930–1035 W microwave heating resulted in bitumen oxidation comparable to RTFOT + PAV aging protocol. In our research, dielectric heating was applied to the 500 g neat bitumen sample poured into 1000 mL quartz glass beaker (0A + M sample). So the prepared specimen was put into a microwave chamber and heated at 800 W @ 2.450 GHz for 900 s (15 min). The microwave heating conditions were chosen from practical reasons as an ultimate time conditions in this study. 1R + M specimens were subjected to microwave heating after pouring bitumen from RTFOT glass cylinders to one quartz glass beaker (ca. 500 g). 1P + M specimens were subjected to microwave heating after pouring bitumen from PAV stainless steel pans to one quartz glass beaker (ca. 500 g) in the original PAV dishes. The testing plan is presented in Table 7. 

### 2.5. Microwave Applicator

A microwave applicator was designed and constructed in the scope of NGAM2 project [44]. The technical details were presented in the recent publication [43]. In this study, the unique real-scale microwave applicator was designed and installed on a trailer, which was towed by a car. The m-w applicator was supplied with the electricity from the portable power generator. Construction details, as well as technology steps, weren’t publicized, as they are protected know-how for the scientific-industry consortium. Generally, the designed applicator had a total microwave nominal power of 7 kW at 2.450 GHz. The design of the applicator consisted of sets of waveguides and microwave generators, whose geometry was adjusted to the linear heating capability, considering linear joint-forming technique and crack healing.

### 2.6. Microwave Assisted Processes

**Figure 4:**
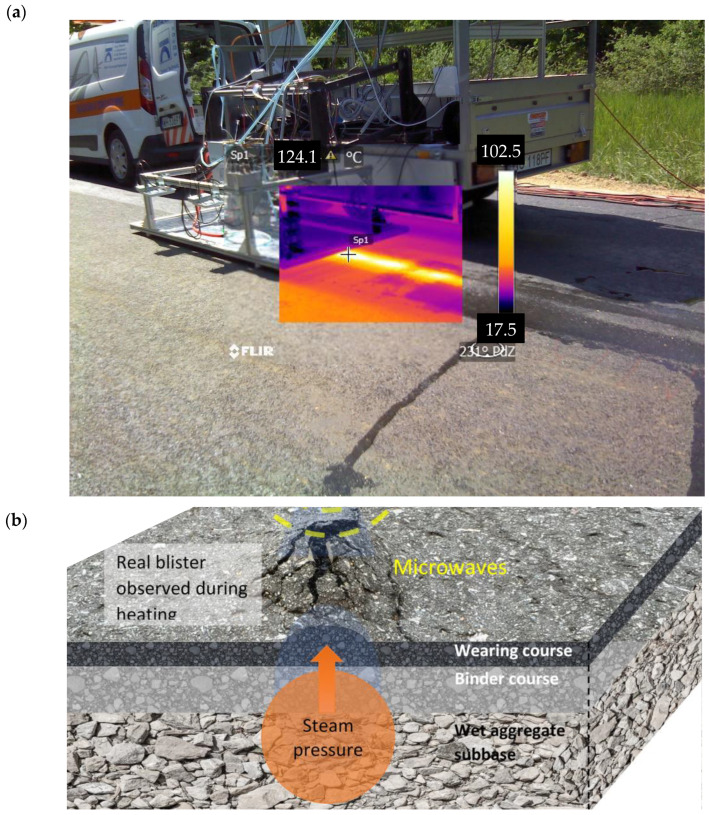
(**a**) Thermal effect of pavement heating using microwave applicator; (**b**) Blistering phenomenon during heating of asphalt.

The joint-forming microwave-assisted process goal was to achieve pavement temperature on both sides of the joint of ca. 140 °C, what enabled the extra compaction process. After pavement joint heat-up on both lanes (cold and hot lane), it was possible to achieve extra compaction process. The heat-up process, which had an initial temperature of ca. 80 °C (hot lane) and ca. 40 °C (cold lane) took from 1 min/1 m (basalt aggregate) to 10 min/1 m (limestone aggregate). There was a known phenomenon occurring during the application of microwave, the rise of asphalt pavement due to the sudden rise of steam pressure. This phenomenon had been observed since the beginning of asphalt treatment using microwave energy. In general, it helps to achieve the assumed goal, the additional compaction of pavement. After the pavement has risen it was rolled using steel-roller compactor and the pavement surface was levelled before cooling down. Both, the effect of joint heating and the effect of blistering, are presented in Figure 4.

In addition to the longitudinal joints evaluation experiment, there was also a trial of cracked pavement repair using microwave applicator performed. The experiment was done on cracked, two-layered asphalt pavement, made of the asphalt concrete (AC 11 B and AC 16 B), 5 + 5 cm thick, with limestone aggregates, paved over the limestone crushed aggregate unbound base. The pavement was built on the crushed stone aggregate base course. The crack in the asphalt layers was a full-depth type (10 cm thick), possibly induced by thermal shrinkage of the pavement. Before microwave treatment, the crack was vacuum cleaned and blown with 10 bar air pressure lance. After the preparation process, the microwave applicator was engaged and heated the pavement in the close vicinity of the crack. The heating process ended up with the pavement surface temperature of ca. 140 °C. The evaluated average process speed was ca. 10 min/1 m of the crack. Due to the blistering phenomenon described later, the pavement moved up and had to be roller compacted to bring the initial surface level. While there are options of remixing the heated asphalt layer or homogenization, no additional activity was applied in this process. 

## 3. Results

### 3.1. General

The test results were grouped by the test methods. Statistical analysis of the results was based on confidence intervals for the mean at a 90% confidence level. Final evaluation of differences between test results was based on ANOVA and Tukey’s paired comparisons (unless otherwise stated). To save space, each presented statistic shows only the MW joint type compared to other joint types. Experimental errors were published in terms of the confidence intervals (CI) provided in the figure bars with the probability of 90%. All tables include Difference of levels, Difference of means, Standard Error (SE) of difference and 90% Confidence Intervals (CI). Tables also contain the adequate statistics: T-values and Adjusted *p*-values. 

### 3.2. Longitudinal Joints

#### 3.2.1. Low-Temperature Cracking TSRST

**Figure 5:**
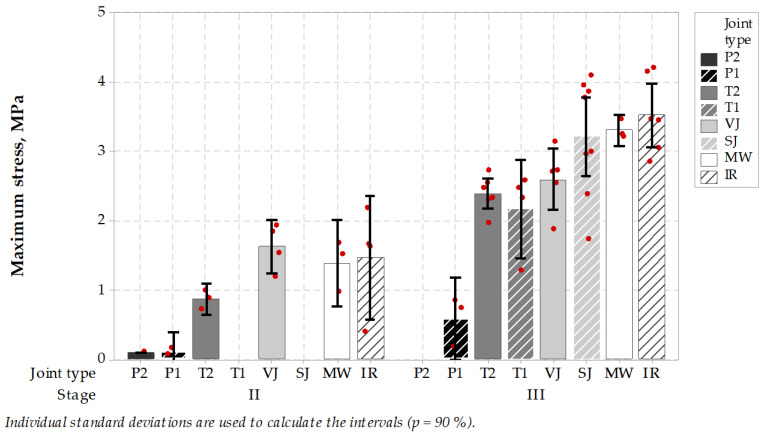
Test results of the maximum stress induced in specimens taken from longitudinal joints (the higher, the better).

**Figure 6:**
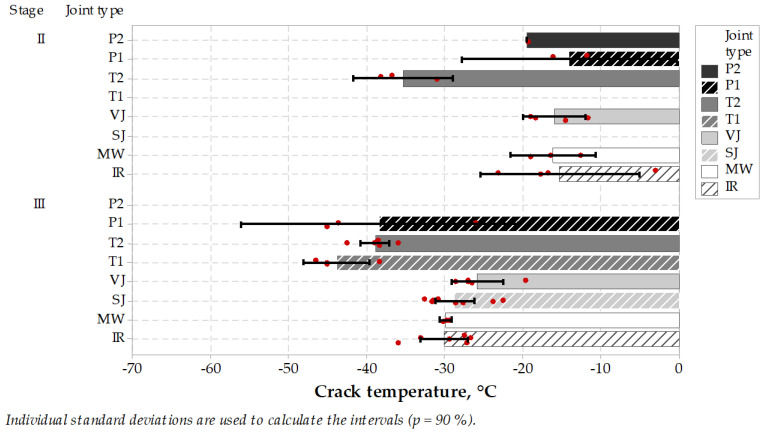
Test results of cracking temperature of specimens taken from longitudinal joints (the higher, the better).

TSRST test results (Figure 5 and Figure 6) showed on one hand that microwave MW longitudinal joint together with other heated (IR) or untreated (VJ) joints performed very well as regards ultimate stresses, reaching a level of 3–4 MPa, compared to 2–3 MPa or less on sealed type joints. On the other hand, the ultimate temperatures during the test were also at a very good level (ca. −15 °C at stage II or −30 °C at stage III), but showed worse performance compared to some of the sealed type joints like T1 and T2 or P1 and P2. An interesting behavior of paste was observed that by relatively low maximum strength at the cracking point, they gained very high elongation and cracked in temperatures reaching −40 °C or even less. Generally, stage II test results showed worse performance than stage III, but it can be explained by different asphalt mixes used in the binder and wearing course, as well as different thicknesses of the layers. Also in stage III, there was a bigger specimen population, and contractors do pay more attention to wearing courses construction. Tukey’s pairwise comparison is presented in Table 8 and Table 9.

It can be concluded that the microwave-heated joint characterized itself with good performance in TSRST. It showed no negative influence comparing to other heated or untreated joint types. In stage III, microwave joint type was proven to have very narrow results range, which can be interpreted as increased uniformity of the joint area in comparison with other jointing techniques. Other jointing techniques gained wider ranges of results or worse mean value of cracking temperatures. It can be caused by lower uniformity of the joint-forming process resulting in higher air voids content, worse uniformity and integrity of the joint and the asphalt layer. Vertical joints, VJ, or skewed joints, SJ, are mostly subjected to errors causing nonuniformity, which is partly reduced using extra heating by microwaves or infrared heating. From these two heating methods, microwave heating is advantageous over infrared heating as a result of its volumetric nature. Infrared heating penetrates from the surface to the depth of heated media, and it results in inadequate uniformity, as is the case of microwave heating. 

In the case of sealed joint types, it can be noticed that better cracking temperatures together with lower ultimate stresses were achieved. It is caused by good properties of sealing materials, but there is a need for the utilization of extra material. Sealing materials are compounded to achieve a very wide spectrum of working conditions, but a non-renewable material and an extra process have to be incorporated into the road construction process. 

Still, the −30 °C TSRST cracking temperature in case of a wearing course joint treated by microwaves, or −15 °C temperature in case of a binder course treated by microwaves, assures high potential of low-temperature cracking resistance of the formed joint. Low cracking temperature is especially important in the cold climate areas, where joints are especially subjected to cracking and premature deterioration.

#### 3.2.2. Indirect Tensile Strength ITS

**Figure 7:**
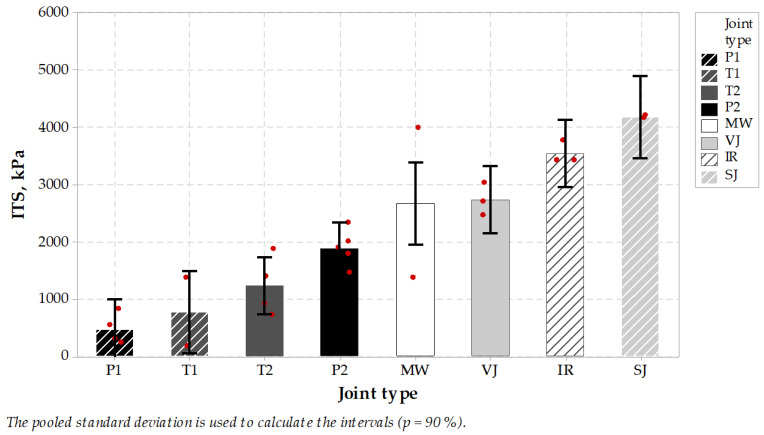
Indirect tension strength test results of specimens taken from longitudinal joints.

**Figure 8:**
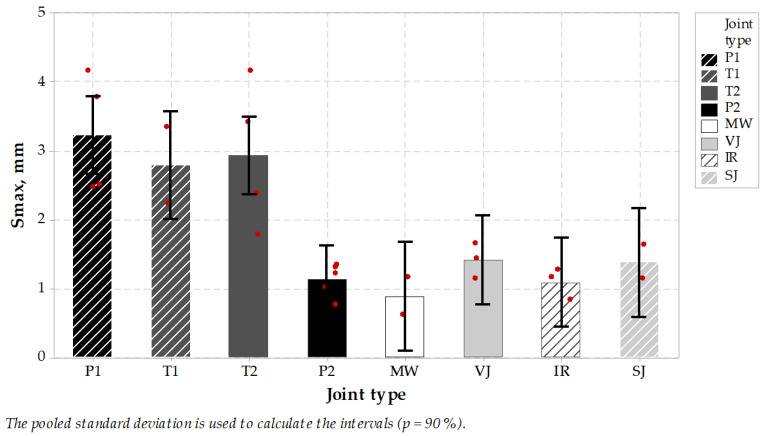
Indirect tension maximum elongation test results of specimens taken from longitudinal joints.

It was a relatively hard task to core 100 mm diameter cores directly from longitudinal joints, where the joint should be located directly in the diameter area of the specimen to have successful test results. Thus not all specimens taken from the pavement could be used in ITS test, and they were rejected. Relatively small specimen population was the reason for evaluation of test results from stage II and III joined in one group. Test results (Figure 7 and Figure 8) were divided into two main groups, the first with results showing high stresses together with low ultimate strain and the second group with low stresses and high strains. Microwave joint type was placed in the middle of indirect tension results population with ca. 2.7 MPa result, compared to 2.7–4.0 MPa for heated (IR) or untreated joints group (SJ or VJ). Also, the maximum elongation reached by the specimens from treated or untreated joint groups and P2 sealed joints were statistically indifferent from the MW-treated joint technique, while sealed P1, T1 and T2 joint techniques were significantly higher.

**Figure 9:**
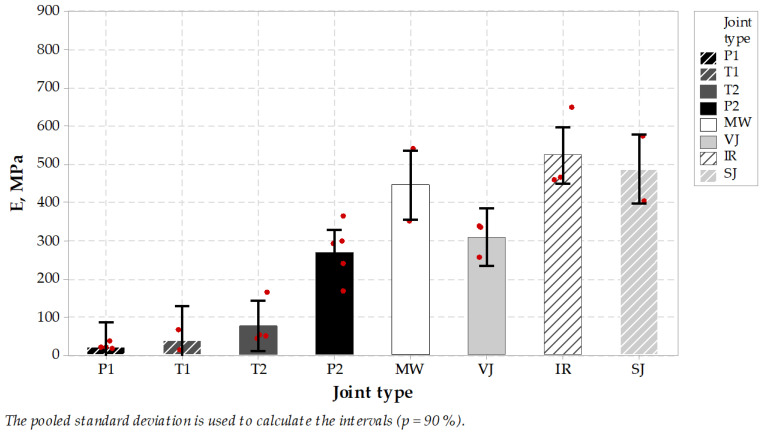
Indirect tension modulus of specimens taken from longitudinal joints.

Next, the E modulus was calculated as a proportion of maximum ITS stress (kPa) to the ultimate strain value (%). It represents the stiffness of the joint and can be concluded just as an indicator in the design process of pavement joints (Figure 9). In this comparison, the MW-treated joint reached an average of 446 MPa, which places this joint type together with IR and SJ in the group of highest results in this comparison. It is worth mentioning that high stiffness value is believed to influence pavement behavior in low temperatures, while low stiffness properties of the joints with sealing material are meant to dilate and to seal the pavement without the possibility of utilizing its properties in pavement response. It may be concluded that MW-treated longitudinal joint test results were positive and placed this jointing technique among other untreated or treated techniques, while sealed joints characterized themselves with lower modulus and higher elongation during testing. Tukey’s pairwise comparison is presented in Table 10 and Table 11.

To summarize this stage of the testing plan it should be emphasized that mid-temperature (0 °C) strength is not less important than low-temperature, sub-zero performance. Test results showed that the microwave joint heating technique resulted in overall performance comparable to infrared joint heated technique. The range of test results was similar as regards stiffness modulus, tensile strength and ultimate strain. The material type joints, namely the vertical and skewed joints, performed almost the same. On the other hand, the sealing materials in 0 °C became softer and resulted in relatively lower ultimate strength and low modulus. It can be concluded that applied sealing materials, especially P1 and T1, were soft enough to dilate the joint but without playing role in bearing the wheel loads from road traffic. These materials are meant to seal the joint, which is correct, but this solution needs to incorporate an extra material and extra installation process.

In all joint types, the uniformity of ITS test results was not so clear as in the case of TSRST test results, probably due to difficulties with discovering the exact location of the joint, which could affect the test result, together with the smaller size of the testing specimens, which could also affect the test result range. It is worth mentioning that ITS test results at 0 °C for cylindrical specimens of typical binder or wearing course asphalt vary between 4000 and 5000 kPa, so it also can be concluded that SJ joint type test results were very close to those values.

#### 3.2.3. Semi-Circular Bending SCB

**Figure 10:**
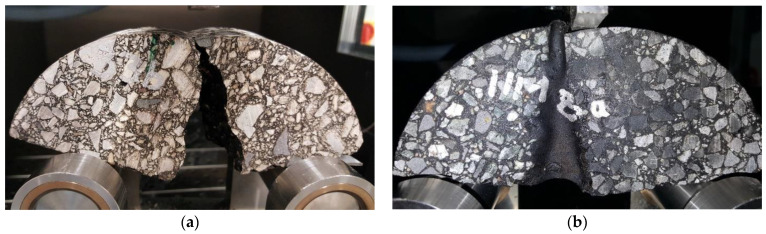
(**a**) Semi-cylindrical specimens after bending test MW; (**b**) Semi-cylindrical specimens after bending test T1.

SCB specimens are cylindrical shaped, 150 mm in diameter. Typically, specimens of this type have an additional notch, the groove that is cut perpendicularly to the specimen diameter and is meant to weaken the specimen cross-section and localize the crack growth location. In the case of specimens cut from the longitudinal joints, this step was omitted, while the joint itself was a weak point of a specimen, and was the most probable location of crack initiation during testing. Example photos of specimens were shown in Figure 10.

**Figure 11:**
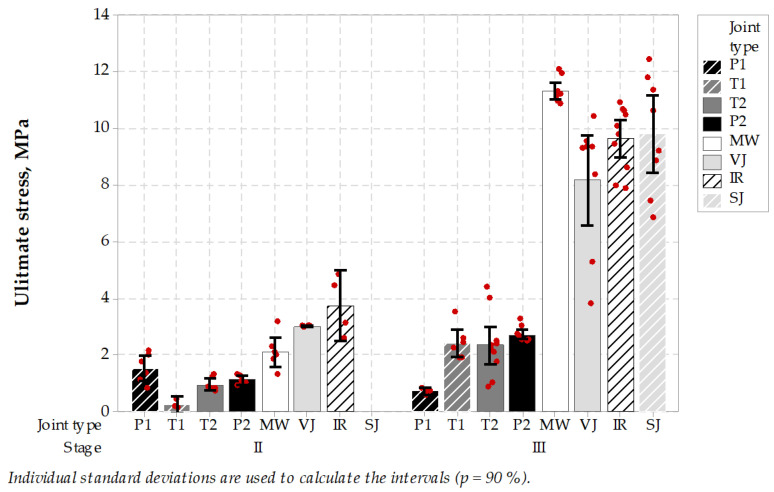
Semi-circular bending test results (the higher, the better).

In the case of stage II of the experiment, untreated and treated type joints performed better than sealed joints. MW joints together with VJ and IR performed better than P2, T1 or T2 while SJ was not present in the test at all, as all specimens failed to reach the testing stage. In stage III MW specimens outperformed rest of specimens, especially P1, P2 or T1 and T2. Results are shown in Figure 11. Tukey’s pairwise comparison statistics are presented in Table 12.

The SCB test protocol uses 150 mm cores. This size of specimen contributed in successful localizing the longitudinal joint in the pavement, thus more specimens were suitable for testing in the laboratory. The microwave-assisted joint showed good performance in stage II tests and an excellent performance in stage III tests. While the variability of test results in stage II was similar in case of all types of unsealed joint construction techniques, in stage III it appeared that the microwave construction technique performed the best in terms of the range of results and ultimate stress level.

Two factors were differentiating the ultimate stress levels presented in stage II and III. These were the differences in asphalt properties used in wearing course and binder course and the presence of sealing material. One can expect that sealed joints specimens, despite representing low stiffness and low tensile strength during SCB tests, were still sealed after the test, which makes the discontinuity in asphalt still waterproof, thus advantageous.

#### 3.2.4. Density Measurements

**Figure 12:**
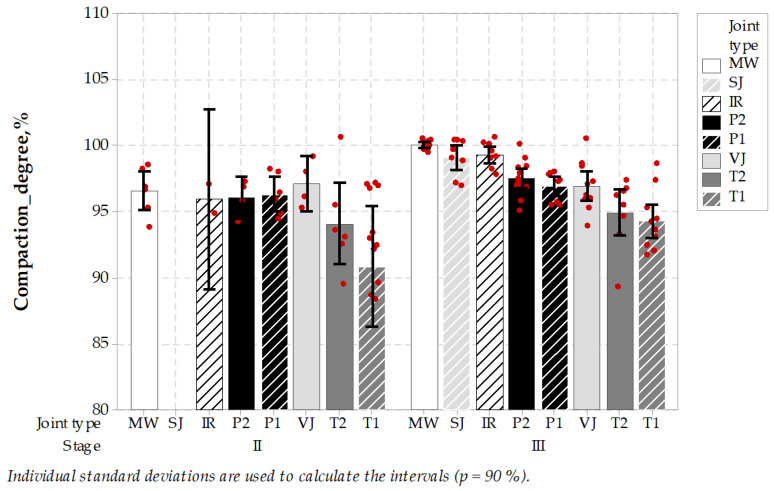
Compaction degree test results of specimens taken from longitudinal joints.

The last part of the evaluation of different joint techniques was the testing of the bulk density of specimens. Measurements were conducted in the saturated, surface dry condition. It can be mentioned that the specimens’ bulk density was measured on specimens that were already tested mechanically and split into half. Both specimen sub-parts were tested individually. The screening concluded on the sub-parts resulted in the rejection of some of the specimens due to the mechanical disintegration. Test results are shown through the compaction degree where the bulk density was divided by bulk reference Marshall density for the mixes and is shown in Figure 12.

The bulk density measurements concluded that in the case of stage II, the average specimen compaction degree for MW, IR, P2, P1 and VJ was in the same statistical group. Lower results for T2 and T1 were obtained, but the differences are not statistically significant. In case of the results from stage III, MW-treated joint results were significantly higher than the results from the other joint types. It can be concluded that asphalt pavement characterized itself with a higher degree of compaction and lower air-voids level comparing to other joint types. The sealing materials used in sealed joint types could lower the density measurements as it was not fully discarded from the specimen and the density of sealing material was significantly lower than the density of the asphalt specimen. But inarguably the MW joint type achieved the highest level of compaction, which is expected from the process of forming of durable joints in the asphalt pavements. Fisher’s pairwise statistics are shown in Table 13.

### 3.3. Crack Repair

#### 3.3.1. General

**Figure 13:**
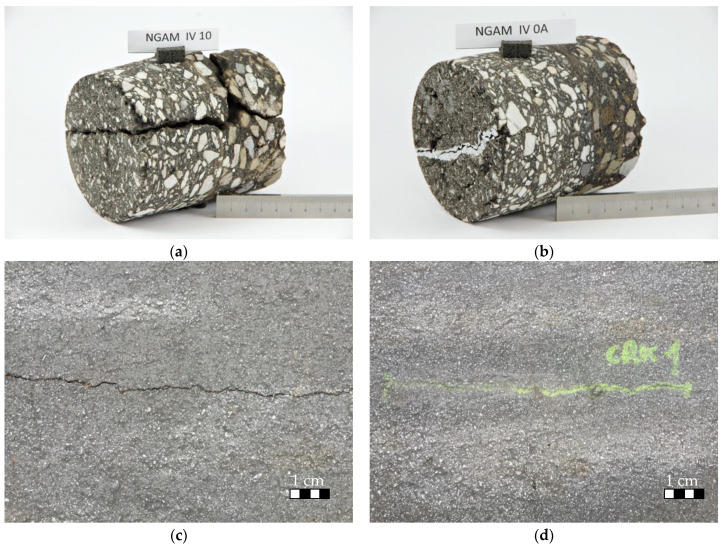
(**a**) Illustration of real-scale test section microwave-assisted asphalt pavement repair (cores were taken from nearby locations), core from the pavement before MW treatment; (**b**) Core from the pavement treated with MW; (**c**) Cracked pavement before MW repair (crack width ca. 5 mm, crack depth–12 cm, full pavement depth, (**d**) Cracked pavement after MW repair.

After finishing the repair process described in Section 2.6, there were also specimens taken from the repaired pavement for ITS and SCB tests. The same statistical assumptions were made for the repaired crack tests. The repair process turned out to be fully successful, as the cracked pavement was healed on its full depth, which is presented in Figure 13.

#### 3.3.2. Indirect Tensile Strength ITS

**Figure 14:**
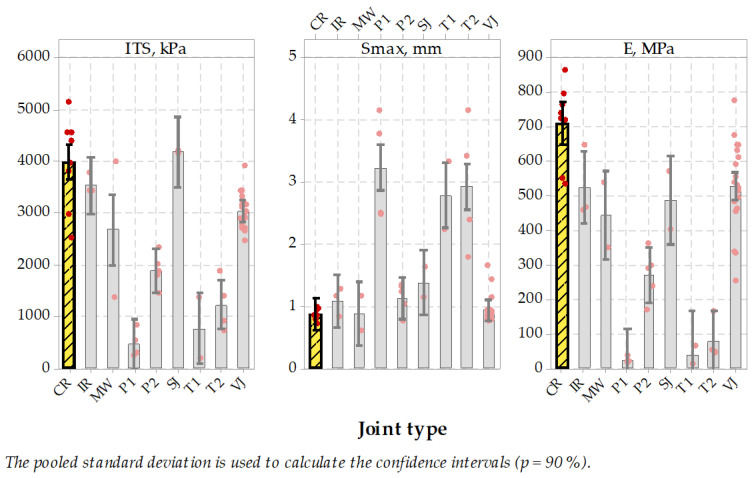
Indirect tension test results on specimens taken from the repaired crack.

All 100 mm specimens were successfully cored from the repaired pavement and they were subjected to indirect tension tests. Obtained results (presented in Figure 14) show that relatively high indirect tensile strength was obtained, almost equal to results obtained on longitudinal joints test section, stage II and III. Also, very high indirect tension modulus was obtained, even of higher value than on stage II and III tests. On the other hand, the ultimate strain was lower than that achieved on specimens from stage II and III, probably because of lower binder content in the tested pavement and absence of polymer-modified bitumen used in mixes from stage II and III.

The formulated statistical hypothesis asked if achieved test results are higher than zero (for cracked pavement). All tests confirmed that achieved mean values of ITS, Smax and E were significantly greater than zero (at *p* < 0.001).

#### 3.3.3. Semi-Circular Bending SCB

**Figure 15:**
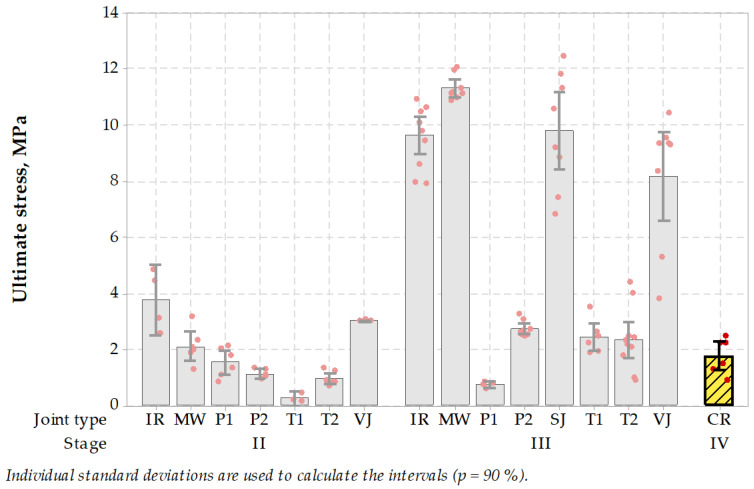
Semi-circular bending test results of the specimens from the repaired crack (CR, stage IV) compared to longitudinal joints (the higher, the better).

Also, all 150 mm cores for semi-circular bending test were successfully taken from the repaired crack area. After cutting the specimens in half, they were tested in the same conditions as specimens taken from longitudinal joints. The results were positive, and an average of 1.8 MPa was achieved (Figure 15). The results were similar to results obtained in stage II of the tests on specimens taken from longitudinal joints. So this experiment was successful. The following statistical hypothesis was formulated: achieved test results are higher than zero (for cracked pavement). The test confirmed that the achieved mean value of ultimate stress was significantly greater than zero (at *p* < 0.001).

#### 3.3.4. Summary of the Crack Repair Process

The described process can be summarized by stating that microwave heating for crack healing is a very promising method. Using this repair technique, there is no need to use either extra sealing materials or perform some noisy cutting operations. The only needed media was the power supply. Every step of this operation was performed with the use of electrical equipment. Considering good, “green” power supply such as solar systems or wind turbines, it can be said that this technology can be described as ecological repair technology. The phenomenon of crack healing was based on the physical process of heating aggregates and bituminous binder, followed by thorough cleaning of the crack faces. The microwave heating made it possible to slightly rearrange the mixture within the area of the cracked asphalt layers and recompact it. The final effect was the regained homogeneity, integrity and stress transferring ability of the asphalt layer. The integrity of the crack made pavement waterproof again, so the possibility of water drainage through the pavement was reduced and deterioration of subbase or subgrade was stopped. The repair process can be repeated in case of additional cracking. Additional sealing materials also could be used and microwave heating could promote the better connection of materials. Further development of this repair method should be continued.

### 3.4. Bitumen

**Figure 16:**
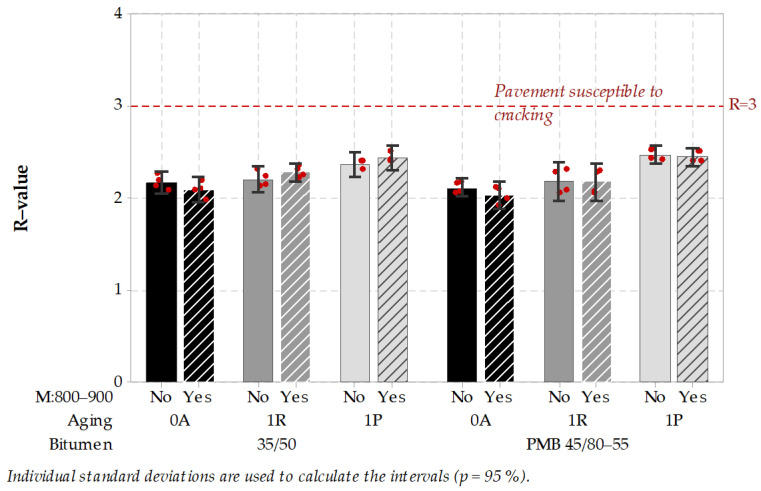
Interval plot of R-value of bitumen obtained in different aging conditions (the higher, the worse).

**Figure 17:**
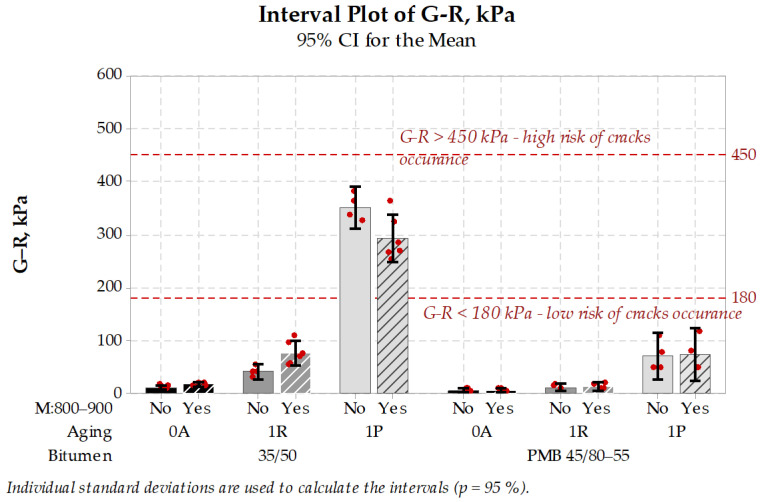
Interval plot of G-R parameter of bitumen obtained in different aging conditions (the higher, the worse).

Test specimens after several stages of aging were tested in Dynamic Shear Rheometer in a wide range of frequency and temperature. After that master curves were built and rheological parameters R-value and G-R parameter were calculated. Calculated parameters are presented in Figure 16 and Figure 17.

The R-value of both tested bitumen were under 3, so none of the tested specimens presented the potential pavement susceptibility to cracking. Changes in R-value following traditional 1R and 1P aging protocols were of significant importance, while the influence of the microwave aging was neither engineering nor statistically important. Higher differences occurred after determining the G-R parameter. Traditional aging protocols influenced G-R parameter of neat bitumen more significantly than PMB. Mostly all cases of aging resulted in G-R parameter not exceeding 180 kPa, which can be interpreted as a low risk of pavement cracking. Only the neat 35/50 bitumen aged according to 1P protocol resulted in a relatively high 350 kPa mean level, and surprisingly after treatment of aged specimens with the microwaves, G-R parameter value was reduced by 50 kPa. According to G-R parameter description, this reduction can be interpreted as decrease of pavement possible susceptibility to cracking. This microwave-induced reduction of G-R parameter should be further investigated on wider range of bitumen types.

To sum up the presented bitumen test results, no negative influence of microwaves on bitumen properties in R-value and G-R parameter was found. Based on these test results, it can be concluded that microwave heating should have no harm on asphalt properties, which could affect the pavement cracking susceptibility. There is even a possibility of reducing the pavement cracking risk with the use of microwave heating, and this finding should be further investigated.

## 4. Discussion

This research was focused on the evaluation of microwave-assisted joint-forming technique in comparison to traditional techniques. Another part of this research was a demonstration of the application of microwave heating technique on the cracked pavement to repair the crack. Joints and repaired cracks were evaluated through mechanical tests and quality tests. Longitudinal joint-forming technique assisted with microwave heating (MW) was placed in the group of other vertical treated or untreated joints, bringing the same or better tensile strength in different temperature ranges. Better compaction of asphalt mixture in the vicinity of longitudinal joints resulted in lower air-voids content and supported the increase of sensitivity to water and frost, homogeneity and flexibility. It also supported the tensile strength increase of the joint, which resulted in good joint performance in around 0 °C and subzero temperatures. In the group of vertical treated joints technique, the microwave heating advances over infrared heating, as it does not affect the bituminous materials the way the heat-transfer based technique does. Microwave energy does not overheat the surface of the bituminous mixture (and bitumen), while the infrared heating is very likely to do so. Comparing the two groups of vertical-treated and vertical-untreated joints to vertical sealed joints, it can be concluded that effectiveness of sealing in both groups was maintained but in a different way. Material sealed joints were softer and less stiff, so they characterized themselves by higher extensibility, while the joint was still sealed. On the opposite side, the treated vertical joints made pavement almost seamless, what was confirmed by high tensile strength values, but also low deflection resulting in high modulus values. The most promising finding of the microwave-assisted joint was a better uniformity and compaction of the asphalt layer along the joint. Tested bulk density of specimens and resulting compaction degree were significantly higher than other types of joints. The microwave applicator was able to gain the operational speed of 0.1–1 m/min (depending on the type of used aggregates). The operational speed is the key factor for the further development of the applicator design. Typical operational speeds during asphalt paving reach 60 m/min, so the proper design of the microwave applicator should account for the adequate performance.

In case of microwave-heated cracking repair, the experiment was successful as cracked pavement regained its integrity on full depth and specimens taken from repaired crack were capable of transferring the tensile loads. The success of the crack repair was based on the possibility of volumetric asphalt mix reheating. Reheated asphalt mix characterized itself with the workability high enough to redistribute aggregates and enable the further compaction of the asphalt mix in the crack vicinity. The total depth of crack repair was ca. 10 cm, but numerical simulations presented in publication [43] suggest that depth of 15–20 cm could be easily reached. The crack heating operational speed was in this case 0.1 m/min, so 1 m crack could be repaired within 10–20 min, including recompaction of the pavement.

There was a blistering phenomenon observed during the heating of longitudinal joints and cracks healing. The phenomenon was significantly more intense on older pavement, which seemed to be more saturated with water compared to a new dry pavement structure.

There are also some challenges of microwave heating to be considered. It must be stated that not every road material is susceptible to dielectric heating in the same way. Even in this research project tested materials characterized themselves with varied reaction to microwave heating, thus resulting in the different time needed to heat the asphalt layer to a suitable temperature. Further research should be concluded to achieve optimum power/time dielectric heating parameters. 

## 5. Conclusions

It can be concluded that microwave heating is a promising method of assisting the construction or repair process of pavement. It does not need to use additional sealing materials, and it can be powered by a green source of energy. The following conclusions were stated in the article:

- MW joints were durable in TSRST test, no negative influence on low-temperature cracking was concluded; even though the maximum stresses were higher than in case of sealed joints, crack temperature level was still on a satisfactory level,
- MW joints were in the middle class for the ITS test, while E modulus presented the highest level in the test set,
- MW joints were significantly in the best class of the ultimate stress test results as regards the wearing course (stage III); it showed an overall significant advantage over material type joints while being the best in the class of the treated and untreated joints,
- the density measurements showed most important MW joint technique advantage over other tested techniques, presenting the highest average value and lowest range of single results; the difference was not significant, but one of the reasons was high standard deviations achieved in case of the other jointing techniques,
- MW crack repair technique brought good results should be observed in a longer period; what is important, the repair process can be repeated according to needs,
- Bitumen tests did not bring any negative conclusions as regards bitumen properties and their predicted influence on cracking susceptibility of asphalt pavement.

The microwave-assisted pavement construction process is a promising way of road works development. The sealing process itself could be easily incorporated and adopted in standard paving equipment. In this article, it was concluded that microwave heating resulted in the formation of longitudinal joints that were durable and mechanically resistant in a wide range of temperatures. What was most important, the pavement in the close vicinity of the microwave-heated joint characterized itself with higher density and compaction level, compared to other joint-forming techniques. This very promising observation could be a significant improvement over standard joint formation techniques and should be further tested. It should be also mentioned that no additional material has to be used and no non-renewable resources were needed. This technique should be tested on a wider scale in real roadwork conditions. In the time of CO_2_ emission reduction needs, any technique that potentially does not need to use non-renewable energy should be considered for use. The first trials of using the microwave technique as a heat source were considered in early 1970s, developed in the ’80s, further tested in ’90s but never came to regular use. If we consider that electrification comes to our everyday life, the microwave heating solutions have never been so close for implementation and commercialization.

## Figures and Tables

**Table 1:** Main groups of pavement longitudinal joints forming technologies.

Technology Group	Brief Description	Advantages	Disadvantages
Seamless asphalt paving	A single paver lays a single layer	Seamless technology, no discontinuities, no compaction issues	Limited by a width of a paver
Hot by hot (echelon paving)	More than one paver lays more than one layer; pavers work side-by-side and overlap	Joints are formed in hot material, the best possible adhesion between layers and compaction within the joint area is kept	Difficulties with paving synchronization, compaction order and homogeneity; multiple sets of paving and compacting equipment needed
Hot by cold	Paver lays asphalt layer and after cooling down the next layer is paved	Work can be staged; different solutions for joints forming are available, using standard equipment, special equipment or special sealing materials	Seams are formed between hot and cold asphalt mix, not optimum adhesion conditions are achieved; mistakes in joint-forming process are frequent; additional material is used

**Table 2:** Evaluated joint construction technologies and crack repair.

Group	Description	Sub-Group	Abbr.	Illustration
Untreated	This technology is commonly practiced when the treated group is not used; it can be considered as a reference technology in this experiment. It comprises two subgroups.	skewed joint (formed with the use of plate attached to roller compactor); slope of 60° in asphalt layer is formed; next hot-paved layer can be placed by cold lane with a negative slope	SJ	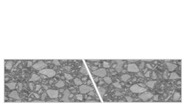
		vertical joint (cut-off-on the fresh mix, with a knife attached to roller compactor); the vertical edge is formed; next hot-paved layer can be placed by cold lane with a vertical edge	VJ	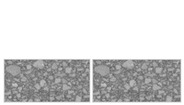
Treated	This technology is neither commonly known nor frequently used; it can be treated as an experimental and innovative group	vertical joint (as above) followed by infrared gas burner used for heating of joint; Infrared (IR) heating can be applied before, during or after next hot-paved layer is placed	IR	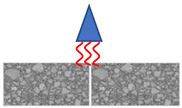
		vertical joint (as described in VJ subgroup) followed by microwave applicator used for heating of joint; Microwave (MW) heating can be applied before, during or after next hot-paved layer is placed	MW	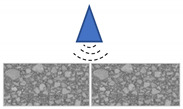
Sealed	This technology is most commonly used; there are additional thermoplastic, bitumen-based materials applied to seal the vertically performed joint	vertical joint (as described in VJ subgroup) followed by application of thermoplastic bituminous sealing tapes before placing of next hot-paved layer	T1 T2	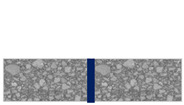
		vertical joint (as described in VJ subgroup) followed by application of bituminous sealing paste: organic solvent-based bitumen emulsion based before placing of next hot-paved layer	P1 P2	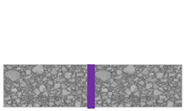
Crack repair	Crack was repaired using microwave heating technique	microwave applicator is used to reheat the cracked pavement; additional operations can be added before (e.g. cleaning) or after (remixing, compaction) the microwave treatment	CR	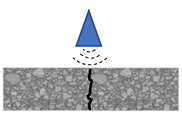

**Table 3:** Asphalt mix design used in the project.

Mix Designation	SMA 8 G	SMA 8 B	AC 16 W	AC 16 B
Aggregate Type	100% Gabbro	100% Basalt	100% Limestone	100% Basalt
Sieve Size, mm (Square Opening)	% Mass	% Mass	% Mass	% Mass
22.4	−	−	100.0	100.0
16	−	−	97.9	98.4
11.2	100.0	100.0	77.3	76.3
8	96.6	96.8	60.8	59.4
5.6	59.7	55.0	46.9	46.2
2	29.5	27.7	24.9	27.7
0.5	−	−	13.3	13.3
0.125	12.3	12.5	7.5	8.1
0.063	11.0	10.0	6.5	6.6
Binder	7.3	6.7	4.3	4.6
Binder type	PMB 45/80–55	PMB 45/80–55	35/50	35/50
Max density, Mg/m^3^	2.601	2.566	2.528	2.692
Bulk density ssd, Mg/m^3^	2.530	2.479	2.414	2.525
Voids content, % *v*/*v*	2.7	3.4	4.4	6.2
Dielectric constant ε′	5.76–6.04	6.45–6.74	6.51–7.01	6.60–7.22

**Table 4:** Stages of experiment.

Stage of the Experiment	Location of Joint	Jointed Mixes Types
II	the joint between binder courses hot by cold	AC 16 W–AC 16 B AC 16 W–AC 16 W
III	the joint between wearing courses hot by cold	SMA 8 G–SMA 8 B SMA 8 B–SMA 8 B
IV	crack repair using microwaves	AC 11–AC 11

**Table 5:** Characteristics of sealing materials.

Material Designation	T1	T2	P1	P2
Material Type	Bituminous Sealing Tape	Bituminous Sealing Tape (Self-Adhesive)	Bituminous Sealing Paste (Solvent-Based)	Bituminous Sealing Paste (Emulsion-Based)
Cone penetration, 0.1 mm	49.6	43.0	32.3	40.0
Softening point, °C	>150	113.9	>150	>150
Flow resistance, mm	0.0	0.0	0.0	0.0
Elastic recovery, %	11	20	14	10
Frost resistance, balls uncracked	4	4	4	4
Continuous extension				
-max. elongation, %	≥10	≥10	≥10	3
-max. force, kN	5.43	13.74	4.10	8.27
Type of break at 10%	No break	No break	No break	Cohesion break
Cold bending (temp.)	No cracks	No cracks	–	–
	−20 °C	−10 °C		
Discontinuous extension at −10 °C				
-%	33	33	33	33
-max. tension stress, MPa	0.2	0.6	1.5	1.0

**Table 6:** Description of testing methodology and specimens.

Test Method	Test Protocol	Specimen Size, Type	Test Conditions
**Mechanical Tests**			
Indirect tensile strength ITS (split tension test)	PN-EN 12697-23, met. A	100 mm cylindrical, cored from the pavement	0 °C
Bending tensile strength SCB (semi-circular bending)	PN-EN 12697-44, no cut	150 mm half-cylindrical, cored from the pavement and cut in half	0 °C
Direct tension TSRST (thermal stress restrained specimen)	PN-EN 12697-46, TSRST	40 mm × 40 mm × 200 mm beam, cut from 100 mm × 100 mm × 200 mm slab cut from pavement	(+10)–(−40) °C (Δ = −10 °C/h)
**Physical Properties**			
Degree of compaction	PN-EN 13108-21, C.4 PN-EN 12697-6, met. B, 25 °C and D	100 mm cylindrical cores	+25 °C

**Table 7:** Aging plan for tested bitumen samples.

Aging Stages	Description	35/50	PMB 45/80–55
No aging	0A	+	+
No aging + microwaves	0A + M:800–900	+	+
RTFOT	1R	+	+
RTFOT + PAV	1P	+	+
RTFOT + microwaves	1R + M:800–900	+	+
RTFOT + PAV + microwaves	1P + M:800–900	+	+

**Table 8:** Tukey’s pairwise comparison for TSRST test–maximum stress.

Stage	Difference of Levels	Difference of Means, MPa	SE of Difference, MPa	90% CI, MPa		T-Value	Adjusted *p*-Value
II	MW–IR	−0.075	0.355	(−1.130;	0.980)	−0.21	1.000
II	P1–MW	−1.273	0.425	(−2.534;	−0.012)	−3.0	0.096
II	P2–MW	−1.281	0.537	(−2.876;	0.314)	−2.39	0.240
II	T2–MW	−0.52	0.380	(−1.648;	0.608)	−1.37	0.744
II	VJ–MW	0.238	0.355	(−0.817;	1.293)	0.67	0.982
III	MW–IR	−0.222	0.404	(−1.374;	0.931)	−0.55	0.998
III	P1–MW	−2.712	0.467	(−4.042;	−1.382)	−5.81	0.000
III	SJ–MW	−0.083	0.387	(−1.186;	1.020)	−0.21	1.000
III	T1–MW	−1.138	0.437	(−2.382;	0.107)	−2.61	0.163
III	T2–MW	−0.91	0.404	(−2.062;	0.242)	−2.25	0.302

**Table 9:** Tukey’s pairwise comparison for TSRST test–crack temperature.

Stage	Difference of Levels	Difference of Means, °C	SE of Difference, °C	90% CI, °C		T-Value	Adjusted *p*-Value
II	MW–IR	−0.86	4.11	(−13.08;	11.35)	−0.21	1.000
II	P1–MW	2.08	4.92	(−12.52;	16.69)	0.42	0.998
II	P2–MW	−3.34	6.22	(−21.81;	15.13)	−0.54	0.993
II	T2–MW	−19.25	4.4	(−32.31;	−6.19)	−4.38	0.011
II	VJ–MW	0.17	4.11	(−12.04;	12.39)	0.04	1.000
III	MW–IR	0.2	6.59	(−19.09;	19.49)	0.03	1.000
III	P1–MW	−8.54	7.61	(−30.81;	13.73)	−1.12	0.947
III	P2–MW	29.83	7.61	(7.56;	52.10)	3.92	0.009
III	SJ–MW	1.12	6.31	(−17.35;	19.58)	0.18	1.000
III	T1–MW	−14.04	7.12	(−34.87;	6.80)	−1.97	0.515

**Table 10:** Tukey’s pairwise comparison for ITS test strength.

Stage	Difference of Levels	Difference of Means, MPa	SE of Difference, MPa	90% CI, MPa		T-Value	Adjusted *p*-Value
II + III	MW–P1	2206	498	(671;	3742)	4.43	0.008
II + III	MW–T1	1905	575	(132;	3678)	3.31	0.066
II + III	MW–T2	1665	525	(46;	3283)	3.17	0.085
II + III	MW–P2	789	481	(−694;	2273)	1.64	0.722
II + III	VJ–MW	55	525	(−1564;	1674)	0.10	1.000
II + III	IR–MW	860	525	(−758;	2479)	1.64	0.723
II + III	SJ–MW	1506	575	(−267;	3280)	2.62	0.218

**Table 11:** Tukey’s pairwise comparison for ITS test modulus, MPa.

Stage	Difference of Levels	Difference of Means, MPa	SE of Difference, MPa	90% CI, MPa		T-Value	Adjusted *p*-Value
II + III	MW–P1	423.3	62.1	(231.7;	614.8)	6.81	0.000
II + III	MW–T1	407.3	71.7	(186.1;	628.5)	5.68	0.001
II + III	MW–T2	398.2	65.5	(196.3;	600.1)	6.08	0.000
II + III	MW–P2	174.7	60.0	(−10.4;	359.7)	2.91	0.135
II + III	VJ–MW	−136.5	65.5	(−338.5;	65.4)	−2.08	0.462
II + III	IR–MW	78.3	65.5	(−123.7;	280.2)	1.19	0.922
II + III	SJ–MW	41.9	71.7	(−179.3;	263.1)	0.58	0.999

**Table 12:** Tukey’s pairwise comparison for SCB test, ultimate stress.

Stage	Difference of Levels	Difference of Means, MPa	SE of Difference, MPa	90% CI, MPa		T-Value	Adjusted *p*-Value
II + III	MW–P1	7.6	0.6	(6.006;	9.266)	13.3	0.000
II + III	MW–T1	6.5	0.5	(5.025;	7.940)	12.6	0.000
II + III	MW–T2	6.2	0.4	(4.900;	7.406)	13.9	0.000
II + III	MW–P2	5.8	0.5	(4.425;	7.091)	12.3	0.000
II + III	MW–VJ	2.0	0.5	(0.654;	3.328)	4.2	0.002
II + III	MW–IR	0.8	0.5	(0.543;	2.056)	1.7	0.718
II + III	MW–SJ	−1.9	0.7	(−3.890;	0.033)	−2.8	0.113

**Table 13:** Fisher’s pairwise comparison for density test, bulk density SSD, Mg/m^3^.

Stage	Difference of Levels	Difference of Means	SE of Difference	90% CI		T-Value	Adjusted *p*-Value
III	SJ–MW	−0.0423	0.0249	(−0.0838;	−0.0007)	−1.70	0.095
III	IR–MW	−0.0431	0.0249	(−0.0847;	−0.0015)	−1.73	0.088
III	P2–MW	−0.0884	0.0228	(−0.1264;	−0.0504)	−3.89	0.000
III	P1–MW	−0.1041	0.0249	(−0.1457;	−0.0625)	−4.18	0.000
III	VJ–MW	−0.1037	0.0236	(−0.1431;	−0.0642)	−4.38	0.000
III	T2–MW	−0.1525	0.0249	(−0.1941;	−0.1109)	−6.12	0.000
III	T1–MW	−0.1694	0.0236	(−0.2088;	−0.1299)	−7.16	0.000

## Data Availability

Not applicable.

## Appendix A

The appendix contains additional material characteristics, which might be useful for evaluation of the test results. Table A1, Table A2, Table A3 and Table A4 contain parameters of the aggregates used to design the asphalt mix. Table A5 contains parameters of bituminous binders. Bitumen were supplied by LOTOS company.

**Table A1:** Properties of basalt aggregate used in “B” type mixes (results by the producer).

Properties (Standard)	Basalt 0/2	Basalt 2/5	Basalt 5/8	Basalt 8/11	Basalt 11/16
Aggregate size d/D (mm), EN 933-1	0/2	2/5	5/8	8/11	11/16
Aggregate distribution category (mm), EN 933-1	G_c_85	G_c_90/10	G_c_90/10	G_c_90/10	G_c_90/10
Aggregate tolerance category (mm), EN 933-1	G_TC_20	G25/15	G25/15	G25/15	G25/15
Aggregate apparent density (Mg/m^3^), EN 1097-6	ρ_a_ 3.00	ρ_a_ 3.01	ρ_a_ 3.02	ρ_a_ 3.01	ρ_a_ 3.01
Agg. oven dried density (Mg/m^3^), EN 1097-6	ρ_rd_ 2.84	ρ_rd_ 2.89	ρ_rd_ 2.90	ρ_rd_ 2.89	ρ_rd_ 2.89
Agg. saturated surface dried dens. (Mg/m^3^), EN 1097-6	ρ_ssd_ 2.90	ρ_ssd_ 2.93	ρ_ssd_ 2.94	ρ_ssd_ 2.93	ρ_ssd_ 2.93
Shape index (mass %), EN 933-4	−	SI_15_	SI_15_	SI_15_	SI_15_
Flakiness index (mass %), EN 933-3	−	FI_10_	FI_10_	FI_10_	FI_10_
Fines content (mass %), EN 933-1	16	2	2	2	2
Fines quality (methylene blue value), EN 933-9	MB_f_10	−	−	−	−
Bitumen affinity (rotating bottle test, %)	−	6 h–90%	6 h–90%	6 h–90%	6 h–90%
EN 12697-11		24 h–70%	24 h–70%	24 h–70%	24 h–70%
Percentage of crushed and broken surface (mass %),	−	C_100/0_	C_100/0_	C_100/0_	C_100/0_
EN 933-5					
Los-Angeles index, EN 1097-2	−	15	15	15	15
Polishing stone value, EN 1097-8	−	48	48	48	48
Aggregate abrasion value, EN 1097-9	−	10	10	10	10
Micro-Deval index, EN 1097-1	−	15	15	15	15
Resistance to thermal shock, EN 1367-5	−	I_%_1.5	I_%_1.5	I_%_1.5	I_%_1.5
	−	V_LA_5	V_LA_5	V_LA_5	V_LA_5
Water absorption 24 h, EN 1097-6	1.9	1.3	1.3	1.3	1.3
Resistance to freezing and thawing, EN 1367-1	−	0.2	0.2	0.2	0.2
Sand angularity index, EN 933-6	E_cs_38	−	−	−	−
“Sonnenbrand” of basalt, EN 1367-3	−	SB_LA_	SB_LA_	SB_LA_	SB_LA_

**Table A2:** Properties of gabbro aggregate used in “G” type mixes (results by the producer).

Properties (Standard)	Gabbro 0/2	Gabbro 2/5	Gabbro 4/8
Aggregate size d/D (mm), EN 933-1	0/2	2/5	4/8
Aggregate distribution category (mm), EN 933-1	G_c_85	G_c_90/15	G_c_90/10
Aggregate tolerance category (mm), EN 933-1	G_TC_10	G_25/15_	G_25/15_
Aggregate apparent density (Mg/m^3^), EN 1097-6	ρ_a_ 2.93	ρ_a_ 2.99	ρ_a_ 2.98
Agg. oven dried density (Mg/m^3^), EN 1097-6	ρ_rd_ 2.83	ρ_rd_ 2.95	ρ_rd_ 2.96
Agg. saturated surface dried dens. (Mg/m^3^), EN 1097-6	ρ_ssd_ 2.87	ρ_ssd_ 2.97	ρ_ssd_ 2.97
Shape index (mass %), EN 933-4	−	SI_20_	SI_15_
Flakiness index (mass %), EN 933-3	−	FI_20_	FI_15_
Fines content (mass %), EN 933-1	10	1	1
Fines quality (methylene blue value), EN 933-9	MB_f_10	−	−
Bitumen affinity (rotating bottle test, %)	−	6 h–62%	6 h–62%
EN 12697-11		24 h–18%	24 h–18%
Percentage of crushed and broken surface (mass %),	−	C_100/0_	C_100/0_
EN 933-5			
Los-Angeles index, EN 1097-2	−	15	15
Polishing stone value, EN 1097-8	−	55	58
Aggregate abrasion value, EN 1097-9	−	10	10
Micro-Deval index, EN 1097-1	−	10	10
Resistance to thermal shock, EN 1367-5	−	I_%_0.8	I_%_0.8
		V_LA_3.4	V_LA_3.4
Water absorption 24 h, EN 1097-6	1.0	1.0	1.0
Resistance to freezing and thawing, EN 1367-1	−	F_1_	F_1_
Sand angularity index, EN 933-6	E_cs_38	−	−

**Table A3:** Properties of limestone aggregate used in “W” type mixes (results by the producer).

Properties (Standard)	Limestone 0/2	Limestone 0/4	Limestone 2/8	Limestone 8/16
Aggregate size d/D (mm), EN 933-1	0/2	0/4	2/8	8/16
Aggregate distribution category (mm), EN 933-1	G_F_85	G_A_90/15	G_c_90/10	G_c_90/20
Aggregate tolerance category (mm), EN 933-1	G_TC_20	G_TC_20	G_20/17.5_	G_20/15_
Aggregate apparent density (Mg/m^3^), EN 1097-6	ρ_a_ 2.71	ρ_a_ 2.71	ρ_a_ 2.71	ρ_a_ 2.71
Agg. oven dried density (Mg/m^3^), EN 1097-6	ρ_rd_ 2.66	ρ_rd_ 2.64	ρ_rd_ 2.68	ρ_rd_ 2.69
Agg. saturated surface dried dens. (Mg/m^3^), EN 1097-6	ρ_ssd_ 2.68	ρ_ssd_ 2.67	ρ_ssd_ 2.69	ρ_ssd_ 2.70
Shape index (mass %), EN 933-4	−	−	SI_25_	SI_25_
Flakiness index (mass %), EN 933-3	−	−	FI_25_	FI_25_
Fines content (mass %), EN 933-1	3	16	2	2
Fines quality (methylene blue value), EN 933-9	−	MB_f_10	−	−
Bitumen affinity (rotating bottle test, %)	−	−	6 h:65–90%	6 h:65–90%
EN 12697-11			24 h:40–80%	24 h:40–80%
Percentage of crushed and broken surface (mass %),	−	−	C_100/0_	C_100/0_
EN 933-5				
Los-Angeles index, EN 1097-2	−	−	30	30
Polishing stone value, EN 1097-8	−	−	44	44
Aggregate abrasion value, EN 1097-9	−	−	15	15
Micro-Deval index, EN 1097-1	−	−	25	25
Resistance to thermal shock, EN 1367-5	−	−	I_%_0.2–1.0	I_%_0.2–1.0
			V_LA_1–4	V_LA_1–4
Water absorption 24 h, EN 1097-6	1.0	1.0	1.0	1.0
Resistance to freezing and thawing, EN 1367-1	−	−	F_1_	F_1_
Sand angularity index, EN 933-6	E_cs_30	E_cs_30	−	−

**Table A4:** Asphalt mix dielectric measurements at 23 °C, (sequential measurements at f = 1.3, 1.9, 2.2, 2.84, 3.97 and 5.23 GHz), 95% Confidence Interval for the mean.

Mix Designation	AC 8 G	SMA 8 B	AC 16 W	AC 16 B
Aggregate type	100% gabbro	100% basalt	100% limestone	100% basalt
Dielectric constant ε′	5.76–6.04	6.45–6.74	6.51–7.01	6.60–7.22
Dielectric loss factor ε″	0.031–0.044	0.293–0.329	0.033–0.035	0.338–0.391

**Table A5:** Basic bitumen characteristics.

Bitumen Characteristic	PMB 45/80−55	35/50
Penetration 25 °C, 0.1× mm, EN 1426	54	42
Softening point, °C, EN 1427	61.0	53.8
Breaking point, °C, EN 12593	−24	−16
